# The safety of co-administration of Bacille Calmette-Guérin (BCG) and influenza vaccines

**DOI:** 10.1371/journal.pone.0268042

**Published:** 2022-06-03

**Authors:** Paola Villanueva, Ushma Wadia, Nigel W. Crawford, Nicole L. Messina, Tobias R. Kollmann, Michaela Lucas, Laurens Manning, Peter Richmond, Laure F. Pittet, Nigel Curtis

**Affiliations:** 1 Department of Paediatrics, The University of Melbourne, Parkville, VIC, Australia; 2 Infection and Immunity, Murdoch Children’s Research Institute, Parkville, VIC, Australia; 3 Infectious Diseases, Royal Children’s Hospital Melbourne, Parkville, VIC, Australia; 4 Department of General Medicine, Royal Children’s Hospital Melbourne, Parkville, VIC, Australia; 5 Wesfarmers Centre for Vaccines and Infectious Diseases, Telethon Kids Institute, Perth, WA, Australia; 6 Immunisation Service, Royal Children’s Hospital Melbourne, Parkville, VIC, Australia; 7 School of Medicine, University of Western Australia, Perth, WA, Australia; 8 Department of Immunology, Sir Charles Gairdner Hospital, Perth, WA, Australia; 9 Departments of Immunology and General Paediatrics, Perth Children’s Hospital, Perth, WA, Australia; 10 Department of Immunology, Pathwest, QE2 Medical Centre, Perth, WA, Australia; 11 Department of Infectious Diseases, Fiona Stanley Hospital, Perth, WA, Australia; IAVI, UNITED STATES

## Abstract

**Background:**

With the emergence of novel vaccines and new applications for older vaccines, co-administration is increasingly likely. The immunomodulatory effects of BCG could theoretically alter the reactogenicity of co-administered vaccines. Using active surveillance in a randomised controlled trial, we aimed to determine whether co-administration of BCG vaccination changes the safety profile of influenza vaccination.

**Methods:**

Participants who received influenza vaccine alone (Influenza group) were compared with those who also received BCG-Denmark vaccine in the contralateral arm (Influenza+BCG group). Data on the influenza vaccination site were collected using serial questionnaires and active follow-up for 3 months post vaccination.

**Results:**

Of 1351 participants in the Influenza+BCG group and 1418 participants in the Influenza group, 2615 (94%) provided influenza vaccine safety data. There was no significant difference in the proportion of participants with any local adverse reaction between the Influenza+BCG group and the Influenza group (918/1293 [71.0%] versus (906/1322 [68.5%], p = 0.17). The proportion of participants reporting any pain, erythema and tenderness at the influenza vaccination site were similar in both groups. Swelling was less frequent (81/1293 [6.3%] versus 119/1322 (9.0%), p = 0.01) and the maximal diameter of erythema was smaller (mean 1.8 cm [SD 2.0] versus 3.0 cm [SD 2.5], p<0.001) in the Influenza+BCG group. Sixteen participants reported serious adverse events: 9 participants in the Influenza+BCG group and 7 in the Influenza group.

**Conclusions:**

Adverse events following influenza vaccination are not increased when BCG is co-administered.

## Introduction

The emergence of novel vaccines [1,2] and new applications for older vaccines [3–5] means vaccines are increasingly likely to be co-administered. The safety of co-administration (simultaneous administration of different vaccines at separate sites) is important to assess as there might be differences in adverse events following immunisation (AEFI) compared with non-concomitant administration [6–10].

Bacille Calmette-Guérin (BCG) vaccine has immunomodulatory effects that are studied, for example, for the prevention and treatment of allergic and autoimmune diseases [11]. This feature is thought to underpin the off-target protective effects of BCG vaccination against other non-mycobacterial pathogens [12]. These immunomodulatory effects could lead to differences in immunogenicity and AEFI following the co-administration of vaccines.

Using active safety surveillance data from a randomised controlled trial of *BCG vaccination to reduce the impact of COVID-19 in healthcare workers* (the BRACE trial; ClinicalTrials.gov NCT04327206) [13], we aimed to determine whether co-administration of BCG vaccination influences the safety profile of influenza vaccination; specifically focusing on local adverse reactions and serious adverse events.

## Methods

### Setting and participants

Healthcare workers (HCW) were recruited in Stage 1 of the BRACE trial in six hospitals in Australia from March to May 2020. The objectives of the trial were to determine if BCG vaccination reduces the incidence of symptomatic and severe COVID-19 compared with no BCG vaccination. Evaluation of the safety of BCG vaccination in HCWs was a secondary objective. The trial protocol has been published elsewhere [14]. Briefly, participants were randomised in a 1:1 ratio and open-label design to receive BCG vaccine and influenza vaccine (Influenza+BCG group) or influenza vaccine alone (Influenza group). Exclusion criteria comprised any contra-indication to BCG or influenza vaccine [14], BCG vaccine administered within the last year, or any COVID-19-specific vaccine administered.

### Intervention

All participants received a single intramuscular dose (0.5 ml, pre-filled syringe) in the upper arm, of a quadrivalent inactivated influenza vaccine (FluQuadri, Sanofi-Aventis Australia; Fluarix Tetra, GSK Australia; or Afluria Quad, Seqirus Australia). Participant 65 years and older received adjuvanted quadrivalent influenza vaccine (Fluad Quad, Seqirus Australia). Participants randomised to the Influenza+BCG group were vaccinated within the same hour by trained BRACE trial staff with a single dose of BCG-Denmark (AJ Vaccines, Copenhagen), 0.1 ml (corresponding to 2–8 x10^5^ colony forming units of *Mycobacterium bovis*, Danish strain 1331) given intradermally in the contralateral upper arm, using a short (10 mm) bevel needle (25G to 30G). All vaccinators were trained in intradermal delivery of BCG vaccine.

Participants were informed about the normal expected local reaction to influenza vaccination and BCG vaccination, and were instructed to contact study staff if they had any concerns.

### Data collection

Demographic data and details on vaccine administration were collected using REDCap [15]. Information on influenza vaccination site evolution was collected through participant-completed web-based daily questionnaires for 2 weeks post vaccination (vaccine diary), and at 3 months post vaccination (questionnaire). Questions addressed the presence, time to onset, duration, and severity of pain, erythema, swelling and tenderness at the vaccination site. Information on BCG vaccination site reaction was collected in a similar way, and has been published elsewhere [16].

### Active safety surveillance

Participants who reported a potential AEFI either through the questionnaires or by notifying the study team, were actively followed-up by designated safety medical doctors (SMD). Adverse events were recorded on standard REDCap forms. Serious adverse events were discussed with a BRACE expert vaccine safety group for final determination of causality.

### Statistical analysis

Statistical analysis was done using StataIC 14.0 (Statacorp LP, College Station, TX, USA). The cumulative incidence of AEFI in the 3 months post vaccination was calculated among participants who either received influenza vaccine alone (Influenza group) or with BCG within the same hour (Influenza+BCG group), and who provided influenza vaccine safety data. Local vaccination site reactions were categorised according to the Food and Drug Administration Toxicity Grading Scale for Healthy Adult and Adolescent Volunteers Enrolled in Preventive Vaccine Clinical Trials [17]. Local reaction grades at the influenza vaccination site were compared between the Influenza+BCG group and the Influenza group, using Chi square or Fisher exact tests. Duration and onset of local reactions were compared using the Mann-Whitney test. The time to local adverse reactions was also compared using Kaplan-Meier curves with log-rank test.

Ethical approval was obtained from The Royal Children’s Hospital Human Research Ethics Committee (HREC 62586) with reciprocal approvals from all participating sites. All participants provided signed informed consent prior to enrolment.

## Results

### Demographic characteristics

**Fig 1:**
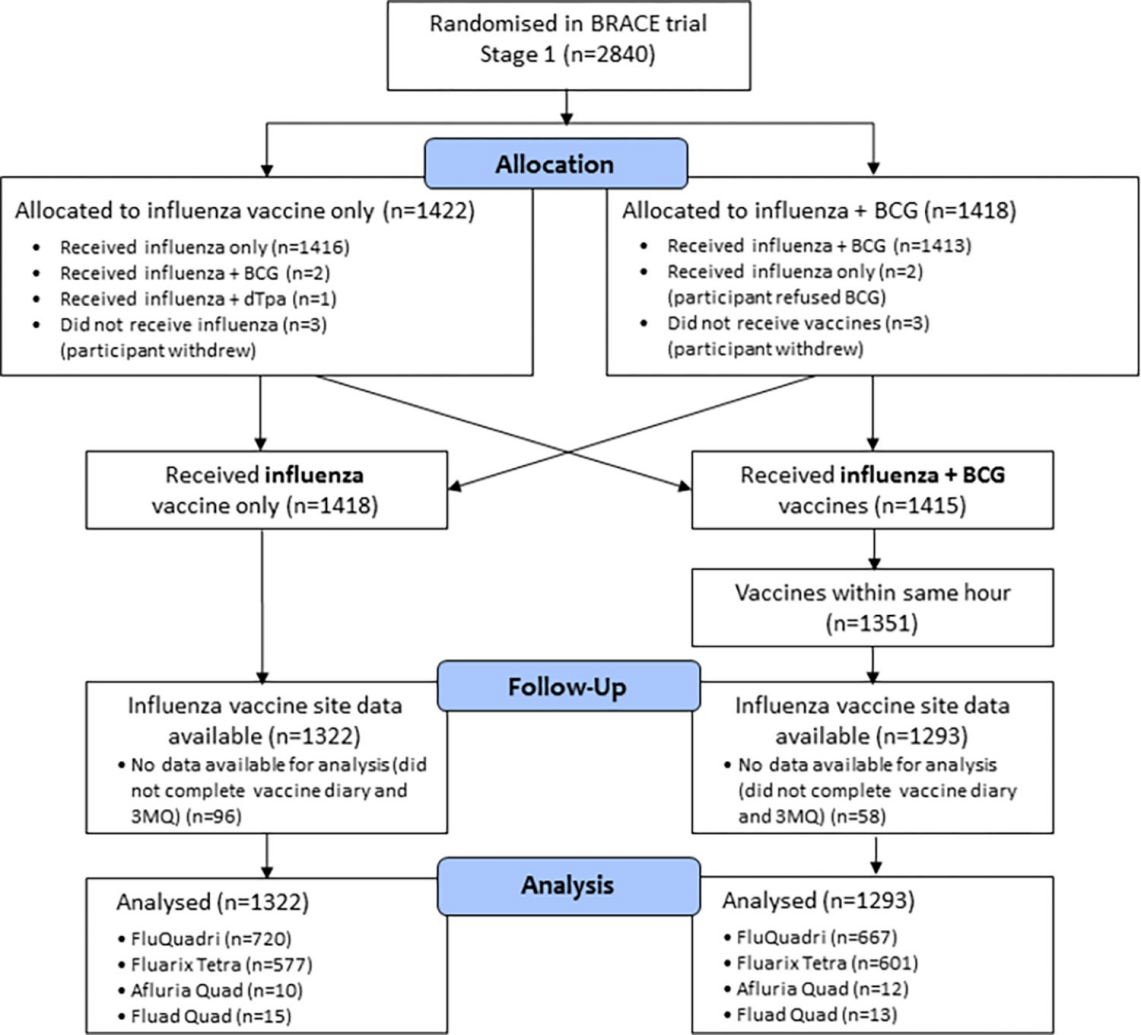
BRACE Stage 1 participant flow. Abbreviations: BCG, Bacille Calmette-Guérin; dTpa, diphtheria-tetanus-acellular pertussis vaccine, reduced antigen formulation; 3MQ, 3-month questionnaire.

Of 1351 participants who were co-administered influenza vaccine and BCG vaccine within the same hour and the 1418 participants who received influenza vaccine alone, 2615/2769 (94.4%) provided influenza vaccine safety data (Fig 1). Demographics and baseline characteristics were similar in both groups (Table 1). The small proportion of participants who were older than 65 years (28/2615, 1.1%) and thus received the adjuvanted influenza vaccine, were equally distributed between both groups.

**Table 1:** Demographics by vaccination group.

	Total	Influenza group	Influenza+BCG group
			
	n = 2615	n = 1322 (%)	n = 1293 (%)
Sex			
Female	2012	1024 (77.5)	988 (76.4)
Male	603	298 (22.5)	305 (23.6)
Age			
Median (IQR)[range] years	40 (31–51) [18–73]	40 (31–51) [19–71]	41 (31–51) [18–73]
State			
Western Australia	1712	853 (64.5)	859 (66.4)
Victoria	903	469 (35.4)	434 (33.6)
Study site			
A	523	271 (20.5)	252 (19.5)
B	693	337 (25.5)	356 (27.5)
C	496	245 (18.5)	251 (19.4)
D	666	352 (26.6)	314 (24.3)
E	215	107 (8.1)	108 (8.4)
F	22	10 (0.8)	12 (0.9)
Role			
Nurse/Midwife	1090	545 (41.2)	545 (42.1)
Medical practitioner	497	254 (19.2)	243 (18.8)
Allied Health	431	223 (16.9)	208 (16.1)
Administrative/clerical	353	173 (13.1)	180 (13.9)
Scientist (medical/research)	95	52 (3.9)	43 (3.3)
PSA/hospital maintenance	127	64 (4.8)	63 (4.9)
Dentist/dental therapy	6	1 (0.1)	5 (0.4)
Other	16	10 (0.8)	43 (3.3)
Influenza vaccine			
FluQuadri	1387	720 (54.5)	667 (51.6)
Fluarix Tetra	1178	577 (43.6)	601 (46.5)
Afluria Quad	22	10 (0.8)	12 (0.9)
Fluad Quad	28	15 (1.1)	13 (1.0)
BCG history at randomisation			
BCG naïve	1268	648 (49.0)	620 (48.0)
BCG previously	1347	674 (51.0)	673 (52.0)

Abbreviations: BCG, Bacille Calmette-Guérin; IQR, interquartile range; PSA, patient services assistant.

### Local adverse events

**Fig 2:**
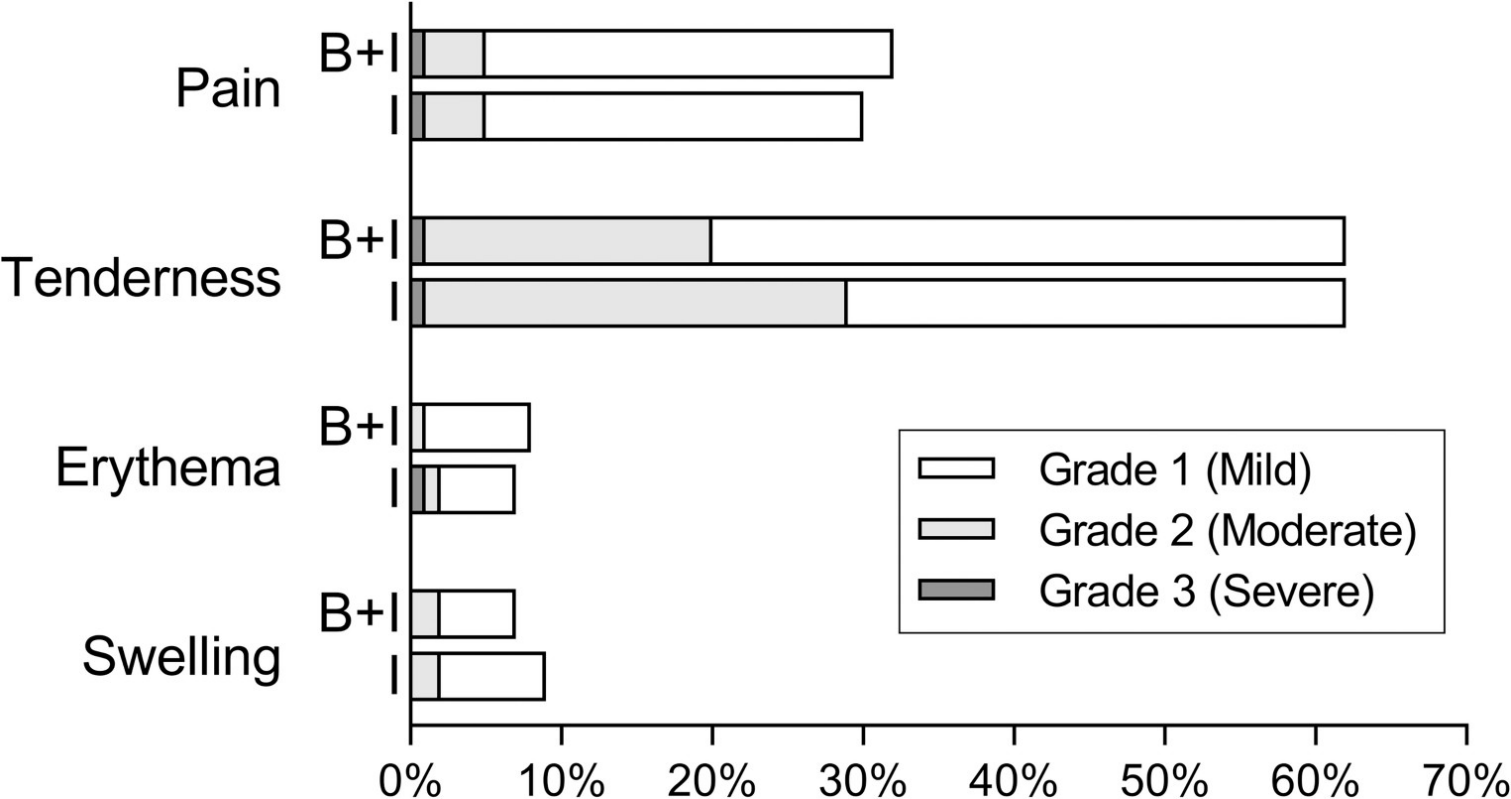
Local adverse reactions at influenza vaccination site. Shown are the proportion of participants in each group reporting at least one of the indicated reactions. Abbreviations: B+I, Influenza+BCG group; I, Influenza group.

Overall, a local adverse reaction to influenza vaccination was reported in 1824/2615 (69.8%) participants, ranging from grade 1 (mild) to 3 (severe) in the categories of pain, tenderness, erythema or swelling at vaccination site (Fig 2). There were no grade 4 (life-threatening) local adverse events.

There was no significant difference in the proportion of participants experiencing any local adverse reaction in the Influenza+BCG group compared with the Influenza group (918/1293 [71.0%] versus 906/1322 [68.5%], p = 0.17) (Table 2).

**Table 2:** Local adverse reactions at influenza vaccination site.

	Total	Influenza group	Influenza+BCG group	p-value
	n = 2615	n = 1322	n = 1293	
**Pain**	781 (29.9%)	384 (29.0%)	397 (30.7%)	0.36
None	1834 (70.1%)	938 (71.0%)	896 (69.3%)	
Grade 1	670 (25.6%)	328 (24.8%)	342 (26.5%)	0.82
Grade 2	108 (4.1%)	55 (4.2%)	53 (4.1%)	
Grade 3	3 (0.1%)	1 (0.1%)	2 (0.2%)	
Grade 4	0 (0.0%)	0 (0.0%)	0 (0.0%)	
Onset, days	1 (1–1)	1 (1–1)	1 (1–1)	0.27
Duration, days	2 (1–2)	1(1–2)	2 (1–2)	0.04
Mean [SD]	1.9 [1.2]	1.7 [1.0]	1.9 [1.3]	
**Tenderness**	1606 (61.4%)	817 (61.8%)	789 (61.0%)	0.68
None	1009 (38.6%)	505 (38.2%)	504 (39.0%)	
Grade 1	971 (37.1%)	434 (32.8%)	537 (41.5%)	<0.001
Grade 2	619 (23.7%)	373 (28.2%)	246 (19.0%)	
Grade 3	16 (0.6%)	10 (0.8%)	6 (0.5%)	
Grade 4	0 (0.0%)	0 (0.0%)	0 (0.0%)	
Onset, days	1 (1–1)	1 (1–1)	1 (1–1)	<0.001
Mean [SD]	1.2 [0.5]	1.2 [0.4]	1.3 [0.6]	
Duration, days	2 (1–3)	2 (2–3)	2 (1–3)	0.87
**Erythema**	173 (6.6%)	79 (6.0%)	94 (7.3%)	0.18
None	2442 (93.4%)	1243 (94.0%)	1199 (92.7%)	
Grade 1	158 (6.0%)	69 (5.2%)	89 (6.9%)	0.13
Grade 2	14 (0.5%)	9 (0.7%)	5 (0.4%)	
Grade 3	1 (<0.1%)	1 (0.1%)	0 (0.0%)	
Grade 4	0 (0.0%)	0 (0.0%)	0 (0.0%)	
Onset, days	1 (1–2)	1(1–2)	1(1–2)	0.02
Mean [SD]	1.5 [0.8]	1.6 [0.9]	1.3 [0.6]	
Duration, days	2 (1–3)	2 (1–3)	2 (1–3)	0.98
Maximal diameter, cm	1.5 (1.0, 3.0)	2.0 (1.0, 5.0)	1.0 (0.5, 2.8)	<0.001
Mean [SD]	2.4 [2.3]	3.0 [2.5]	1.8 [2.0]	
**Swelling**	200 (7.6%)	119 (9.0%)	81 (6.3%)	0.01
None	2415 (92.4%)	1203 (91.0%)	1212 (93.7%)	
Grade 1	157 (6.0%)	97 (7.3%)	60 (4.6%)	0.21
Grade 2	43 (1.6%)	22 (1.7%)	21 (1.6%)	
Grade 3	0 (0.0%)	0 (0.0%)	0 (0.0%)	
Grade 4	0 (0.0%)	0 (0.0%)	0 (0.0%)	
Onset, days	1 (1–2)	1 (1–2)	1 (1–2)	0.59
Duration, days	2 (1–3)	2 (1–2)	2 (1–3)	0.04
Mean [SD]	2.1 [1.3]	2.0 [1.2]	2.3 [1.4]	
Maximal diameter [cm]	2.0 (1.0, 4.0)	2.0 (1.0, 4.0)	2.0 (1.0, 3.0)	0.06
Mean [SD]	2.7 [2.2]	2.8 [2.0]	2.5 [2.3]	

Data are presented as n (%) or median (interquartile range), unless otherwise specified.

#### Pain

**Fig 3:**
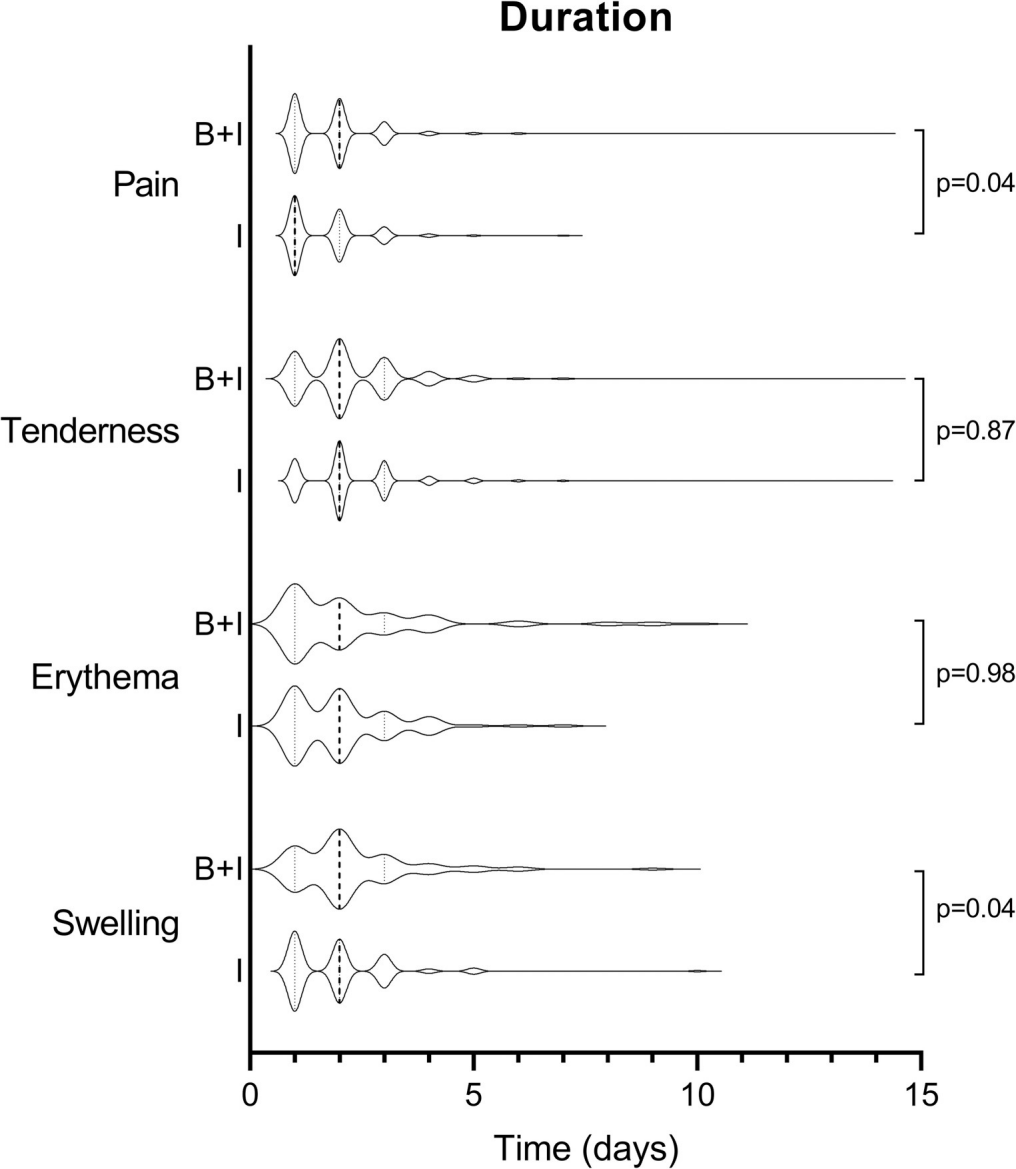
Duration of local reactions at influenza vaccination site, by vaccination group. Dashed lines represent median and interquartile ranges. Abbreviations: B+I, Influenza+BCG group; I, Influenza group.

The proportion of participants experiencing any pain at the influenza vaccination site, time to onset and the distribution of local reaction grades, were similar between both groups (Table 2, Fig 2). The duration of pain was slightly longer in the influenza+BCG group compared with the Influenza group (mean 1.9 days [SD 1.3] versus 1.7 days [SD 1.0], p = 0.04; Table 2, Fig 3).

#### Tenderness

The proportion of participants experiencing any tenderness at the influenza vaccination site was similar between both groups (Table 2, Fig 2). The tenderness grade was lower in the Influenza+BCG group compared with the Influenza group (p<0.001) and occurred later (mean 1.3 days [SD 0.6] versus 1.2 days [SD 0.4], p<0.001; Table 2, Fig 3).

#### Erythema

**Fig 4:**
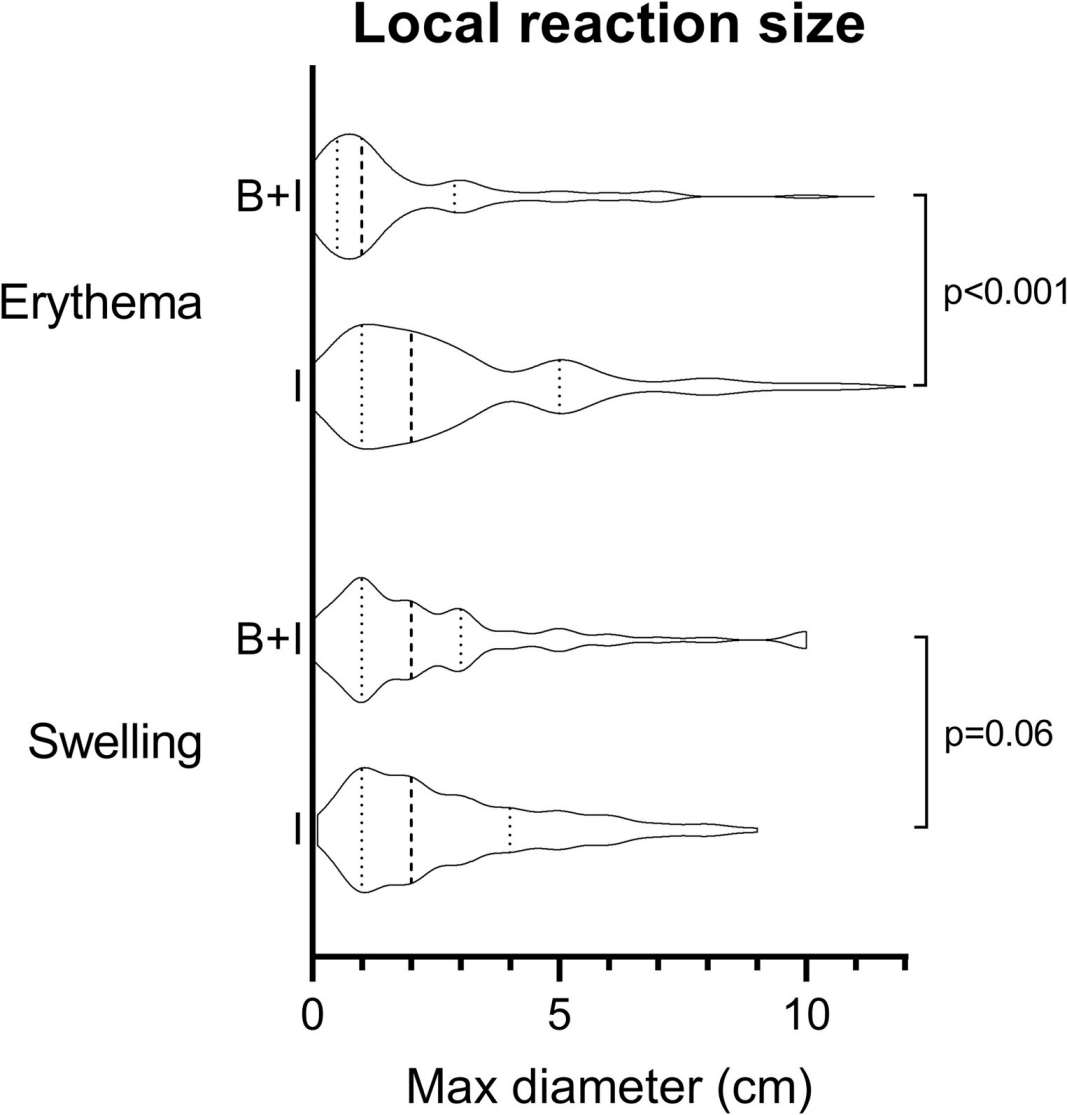
Size of swelling and erythema at influenza vaccination site, by vaccination group. Dashed lines represent median and interquartile ranges. Abbreviations: B+I, Influenza+BCG group; I, Influenza group.

The proportion of participants experiencing any erythema at the influenza vaccination site, as well as the distribution of local reaction grades, was similar between both groups (Table 2, Fig 2). Erythema occurred earlier in the Influenza+BCG group compared with the Influenza group (mean 1.3 days [SD 0.6] versus 1.6 days [SD 0.9], p = 0.02; Table 2, Fig 3) and with a smaller maximal diameter (mean 1.8 cm [SD 2.0] versus 3.0 cm [SD 2.5], p<0.001; Fig 4).

#### Swelling

A lower proportion of participants in the Influenza+BCG group experienced swelling at the influenza vaccination site, compared with the Influenza group (81/1293 [6.3%] versus 119/1322 (9.0%), p = 0.01; Table 3, and S1 Fig). The distribution of local reaction grades was similar between vaccination groups (Table 2, Fig 2). There was a longer duration of swelling in the influenza+BCG group compared with the Influenza group (mean 2.3 days [SD 1.4] versus 2.1 days [SD 1.3], p = 0.04). Maximal diameter of swelling at influenza vaccination site was smaller in the Influenza+BCG group (mean 2.5 cm [SD 2.3], vs. 2.8 cm [SD 2.0], p = 0.06; Fig 4).

**Table 3:** Summary of local adverse reactions at influenza vaccination site in Influenza+BCG group compared with Influenza group.

Influenza+BCG vs Influenza alone	Frequency	Severity	Size	Time to onset	Duration
Pain					↑Increased
Tenderness		↓Reduced		↑Increased	
Erythema			↓Reduced	↓Reduced	
Swelling	↓Reduced		↓Reduced^a^		↑Increased

(^a^p = 0.06).

A sensitivity analysis excluding the 28 participants aged >65 years who received adjuvanted quadrivalent influenza vaccine showed similar findings for pain, tenderness, erythema and swelling.

#### Prior BCG vaccination history

Amongst the Influenza+BCG group, the proportion of participants reporting any pain or tenderness at the influenza vaccination site was less amongst the influenza + BCG-revaccination subgroup compared with the influenza + BCG-naïve subgroup (S2 Table). However, participants who were BCG-naïve were younger compared with the BCG-revaccinated group. Using a regression model, participant age at vaccination appears to be a confounder. When adjusted for age, prior BCG history did not influence pain or tenderness perception at the influenza vaccination site. The younger the age, the more likely the participant was to report any pain or tenderness, regardless of prior BCG history, and remains so when excluding the participants >65 years who received adjuvanted quadrivalent influenza vaccine (n = 13).

### Serious adverse events

Sixteen participants reported serious adverse events (SAE): 9 participants in the Influenza+BCG group and 7 in the Influenza group (p = 0.6). Two participants in the Influenza+BCG group had SAEs that were deemed by the BRACE expert vaccine safety group to be possibly related to the influenza vaccine: 1 hospitalisation 8 hours after vaccination for sudden onset severe headache, myalgia, vomiting and fever, treated with intravenous hydration and anti-emetics; 1 hospital emergency department presentation 10 hours after vaccination for cough and wheeze, treated with nebulised salbutamol. The other 14 SAE were hospitalisations deemed unrelated to vaccination (S1 Table) by the BRACE expert vaccine safety group.

## Discussion

This is the first study to report the safety of co-administration of BCG and influenza vaccines. In a large sample of over 2500 participants and using active surveillance, we found similar proportions of participants experiencing local adverse reactions at the influenza vaccination site when receiving influenza vaccine alone and when co-administered with BCG vaccine on the contralateral arm.

Previous studies have reported on the safety of co-administrating the seasonal influenza vaccine with non-live vaccines such as pneumococcal, herpes zoster or diphtheria-tetanus-acellular pertussis vaccine [18–22]. More recently, the safety of administering a COVID-19 vaccine (NVX-CoV2373) at the same time as an influenza vaccine in adults has been described [23], although this study did not report on local reactogenicity at the influenza vaccine site. One study reported that influenza vaccination may have off-target protective effects against COVID-19 [24].

In our study, a lower proportion of participants in the Influenza+BCG group experienced swelling at the influenza vaccination site compared with the Influenza group. The diameters of the local swelling and erythema reactions around the vaccination site were also smaller, while the onset timing of erythema and tenderness, as well as duration of pain and swelling, were not clinically significant.

The majority of local adverse reactions in each vaccination group were mild (Grade 1) and did not persist beyond 5–7 days. Prior BCG vaccination history did not influence local adverse reactions at the influenza vaccination site when adjusted for age. Younger participants were more likely to report pain or tenderness, regardless of prior BCG history. Other vaccine studies have similarly shown younger age groups reporting pain more frequently at their vaccination site, compared with older age groups [25,26].

There was a low frequency of SAE, consistent with expected AEFI to influenza vaccine [27–29]. Most SAE (7/9) were hospitalisations deemed unrelated to vaccination. The two SAE deemed possibly related to influenza vaccination (1 participant with headache, myalgia, fever and gastrointestinal symptoms; 1 participant with potential asthma exacerbation) are potential AEFI to influenza vaccine [30,31]. However, the causal relationship between influenza vaccination and asthma exacerbation is not clearly established, as influenza vaccination is usually administered during winter, when the background asthma exacerbation incidence is high. Other studies report that influenza vaccination does not increase the incidence of asthma exacerbations compared to placebo [32,33].

Limitations of our study include, firstly, the absence of a placebo injection in the contralateral arm of participants in the Influenza group to control for two injections being given, as this may alter perception of pain and tenderness at the influenza vaccination site. Participants in the Influenza+BCG group may have rated their level of discomfort at the influenza vaccination site relative to any discomfort experienced at the BCG vaccination site. However, the more objective measures of erythema and swelling at the influenza vaccination site in the Influenza group are unlikely to be perceived differently when a second placebo injection is given in the contralateral arm. Secondly, a minority (1.1%) of participants received an adjuvanted quadrivalent influenza vaccine (Fluad Quad), which is preferentially recommended for people aged 65 years and greater in Australia, as it is more immunogenic due to the adjuvant MF59C.1 [34]. Consequently, it has an increased risk of injection site reactions compared with standard quadrivalent influenza vaccine formulations [35]. However, participants receiving this adjuvanted vaccine in the present study were balanced between the Influenza and Influenza+BCG groups, and a sensitivity analysis excluding these participants showed similar results. Thirdly, our study did not assess whether the local reaction at the BCG vaccination site was affected by co-administration with influenza, as no participants were randomised to receive BCG alone. The site of administration of sequential vaccine doses (consistent vs. alternating limbs) has previously been shown to affect vaccine responses [36–38]. One case report of a child who received both BCG and influenza vaccines in close proximity in the same arm, describes significant arm swelling and erythema, erosion and blister formation at BCG vaccination site, on day one post vaccination [39]. The authors hypothesised an antigen–antibody reaction between both vaccines and made a recommendation for subsequent vaccinations to be distant from the BCG vaccination site.

In conclusion, co-administration of BCG and influenza vaccines does not lead to an increase in local adverse reactions or serious adverse events compared with influenza vaccination alone. Future studies should investigate the effect of co-administration on immunogenicity.

## Supporting information

S1 Fig Onset of local reactions at influenza vaccination site, by vaccination group. Kaplan–Meier curves from a time-to-event analysis show estimates of the proportion of participants that did not experience any pain (Panel A), tenderness (Panel B), erythema (Panel C), and swelling (Panel D) at the influenza vaccination site. Abbreviations: B+I, Influenza+BCG group; I, Influenza group.
(PDF)

S1 Table Serious adverse events (SAE). (PDF)

S2 Table Local adverse reactions at influenza vaccination site, by prior BCG vaccination history. Data are presented as n (%) or median (interquartile range), unless otherwise specified.
(PDF)

S1 File Brace trial consortium group list. (PDF)
