# Supplementary material for: The safety of co-administration of Bacille Calmette-Guérin (BCG) and influenza vaccines
Source: PLoS One. 2022 Jun 3;17(6):e0268042. doi: 10.1371/journal.pone.0268042 (PMC9165819; doi:10.1371/journal.pone.0268042)
Supplement: S1 Fig — Kaplan–Meier curves from a time-to-event analysis show estimates of the proportion of participants that did not experience any pain (Panel A), tenderness (Panel B), erythema (Panel C), and swelling (Panel D) at the influenza vaccination site. Abbreviations: B+I, Influenza+BCG group; I, Influenza group. (PDF) [file pone.0268042.s001.pdf]

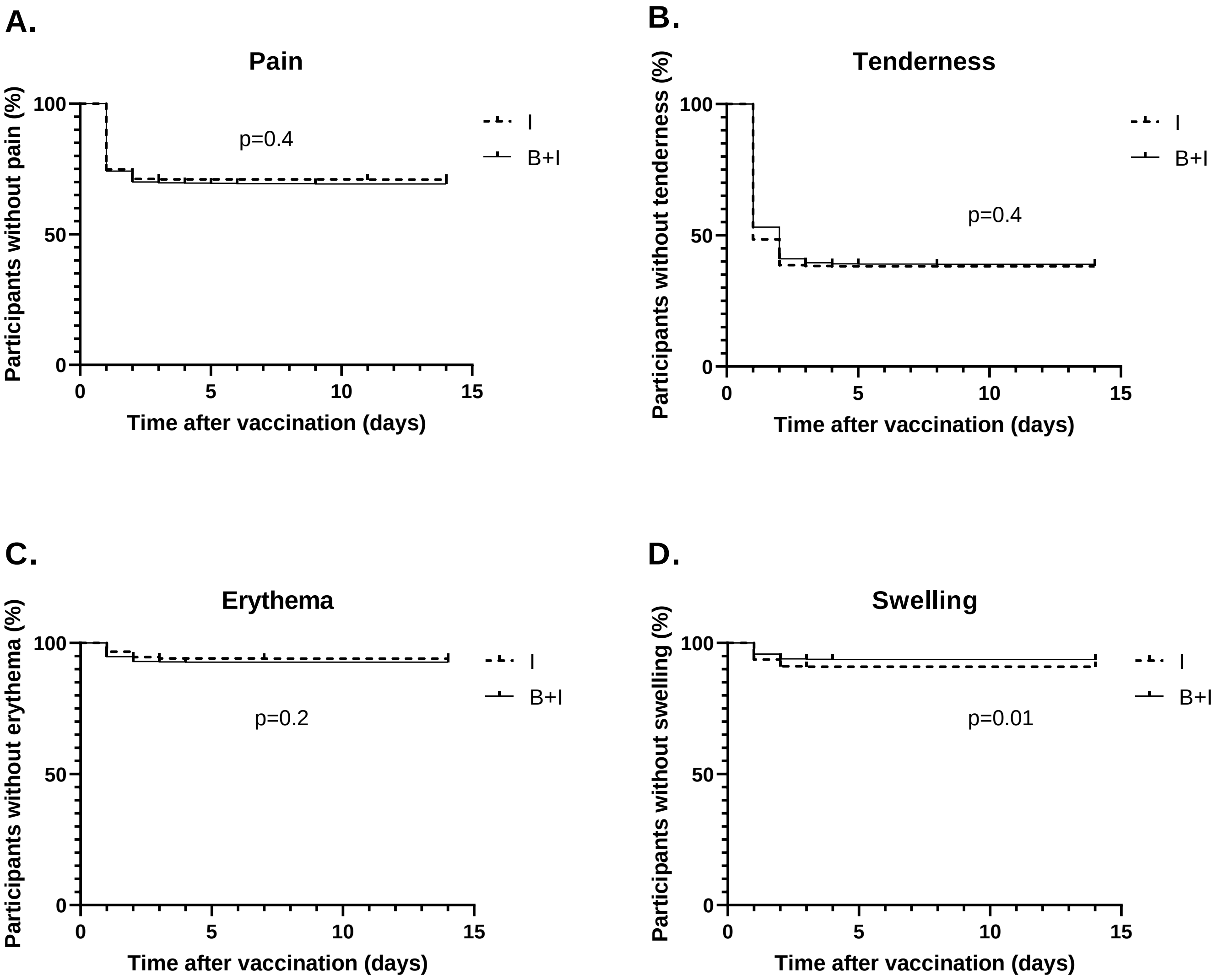

S1 Fig. Onset of local reactions at influenza vaccination site, by vaccination group.

Kaplan–Meier curves from a time-to-event analysis show estimates of the proportion of participants that did not experience any pain (Panel A), tenderness (Panel B), erythema (Panel C), and swelling (Panel D) at the influenza vaccination site.

Abbreviations: B+I, Influenza+BCG group; I, Influenza group
