# Supplementary material for: The safety of co-administration of Bacille Calmette-Guérin (BCG) and influenza vaccines
Source: PLoS One. 2022 Jun 3;17(6):e0268042. doi: 10.1371/journal.pone.0268042 (PMC9165819; doi:10.1371/journal.pone.0268042)
Supplement: S1 Table — (PDF) [file pone.0268042.s002.pdf]

S1 Table. Serious adverse events (SAE)

Participant	Sex	Vaccine group	Onset following vaccination	Description of adverse event	SAE type[a]	Treatment	Relationship to vaccine[b]<br><br>Site Investigator (S) assessment<br><br>MCRI Sponsor (M) assessment	Severity of SAE[c]	Outcome
1	F	Influenza+BCG	8 hours	Sudden onset severe headache ('worst headache in life') then myalgia, vomiting, fever 38.3°C, tachycardia. COVID-19 testing negative. Taken to hospital via ambulance.	3	Hospitalisation overnight: Intravenous paracetamol, ondansetron, metoclopramide, fluid therapy.	(S)(M) Possibly related to influenza vaccine	Severe	Discharged home Day 2
2	F	Influenza+BCG	10 hours	Developed sore throat and felt unwell on evening of Day 1. Day 2 woke up with sore throat, wheeze and cough. Known past medical history of mild asthma. Used salbutamol inhaler at home, with limited effect. COVID-19 testing negative. Worsening cough and wheeze over next few hours led to emergency department (ED) presentation. In ED, afebrile, no sign of respiratory distress or audible wheeze on auscultation. Mild erythematous pharynx.	7	Emergency department presentation: salbutamol nebuliser and 25mg of oral prednisolone. Discharged home within two hours.	(S)(M) Possibly related to influenza vaccine in the context of past medical history of previous mild asthma.	Severe	Discharged home Day 2
3	F	Influenza+BCG	Day 58	Overnight hospitalisation for an episode of pyelonephritis, on a background of chronic urological health issues (long term stoma and indwelling catheter). Frequent urinary tract infections.	3	Hospitalisation overnight: 24 hours of intravenous antibiotics and intravenous fluid therapy.	(S)(M) Unrelated	Severe	Discharged home Day 59
4	F	Influenza+BCG	Day 68	Hospitalisation for shoulder surgery secondary to workplace accident.	3	Hospitalisation: shoulder surgery	(S)(M) Unrelated	Severe	Discharged home Day 70
5	F	Influenza+BCG	Day 71	5-night hospitalisation for abdominal pain with history of diverticulitis. Diagnosed with diverticulitis.	3	Hospitalisation: antibiotics and analgesia	(S)(M) Unrelated	Severe	Discharged home Day 77
6	F	Influenza+BCG	Day 63	Hospitalisation for unmanageable back pain and altered sensation in left foot. MRI spine diagnosis of L5 & S1 nerve impingement and L4, L5, S1 disc prolapse.<br>Past history of fractured back in childhood.	3	Hospitalisation: CT guided nerve block, analgesia and supportive care. Insertion of indwelling catheter	(S)(M) Unrelated	Severe	Discharged home Day 71
7	F	Influenza+BCG	Day 23	2-night hospitalisation for acute appendicitis	3	Hospitalisation: laparoscopic appendectomy	(S) Unlikely<br>(M) Unrelated	Severe	Discharged home Day 25
8	F	Influenza+BCG	Day 23	Hospitalisation for mental health concerns.	3	Hospitalisation	(S)(M) Unrelated	Severe	Discharged home Day 26
9	F	Influenza+BCG	Day 17	Hospitalisation for hand/wrist cellulitis; infected cat bites, multiple puncture marks.	3	Hospitalisation: surgical washout and debridement of injured right hand/wrist and antibiotics.	(S)(M) Unrelated	Severe	Discharged home Day 19
10	F	Influenza	Day 20	Overnight hospitalisation for infected sebaceous cyst on neck	3	Hospitalisation: drainage of cyst and intravenous antibiotics	(S)(M) Unrelated	Severe	Discharged home Day 21
11	F	Influenza	Day 27	4-day hospitalisation for fever, sore throat, headaches, photophobia. Investigated for COVID-19 (negative), blood cultures,	3	Hospitalisation: intravenous fluid therapy for 3 days, antibiotics and	(S)(M) Unrelated	Severe	Discharged home Day 33

				urine, brain imaging, lumbar puncture. Diagnosis: tonsillitis		antivirals. Regular analgesia			
12	F	Influenza	Day 64	Hospitalisation for two nights for fractured ankle requiring internal fixation	3	Hospitalisation: surgery for fractured ankle requiring internal fixation.	(S)(M) Unrelated	Severe	Discharged home Day 66
13	M	Influenza	Day 80	Hospitalisation for sports injury; lower limb fractures requiring surgery	3	Hospitalisation: surgery for lower limb fractures.	(S)(M) Unrelated	Severe	Discharged home Day 82
14	F	Influenza	Day 83	5-day hospitalisation for mental health concerns. Tested negative twice for COVID-19 during hospital stay (respiratory symptoms).	3	Hospitalisation	(S)(M) Unrelated	Severe	Discharged home Day 88
15	M	Influenza	Day 56	2-night hospitalisation for lower abdominal pain due to diverticulitis	3	Hospitalisation: antibiotics and analgesia	(S) Unlikely<br>(M) Unrelated	Severe	Discharged home Day 58
16	F	Influenza	Day 14	Hospitalisation for investigation for iron deficiency and possible inflammatory bowel syndrome; During hospitalisation, fell and hit head, with subsequent seizure. Found to have hyponatraemia, Past history of childhood epilepsy.	3	Hospitalisation: seizure investigations, electrolyte abnormality corrected, prophylactic antibiotics.	(S)(M) Unrelated	Severe	Discharged home Day 17

Note: day 1= day of vaccination

[a]SAE type = criteria for seriousness
1. Resulted in death
2. Immediately life-threatening
3. Requires inpatient hospitalisation (i.e. minimum overnight admission that is non-elective).
4. Results in prolongation of existing hospitalisation
5. Results in persistent or significant disability/incapacity
6. Is a congenital anomaly/birth defect
7. In the medical judgment of the treating physician and/or investigator, it may jeopardise the participant or require intervention to prevent one of the above outcomes

[b]Severity of SAE:
- Severe (severe medically significant but not immediately life threatening)
- Life-threatening (immediately life-threatening)
- Death related to adverse event

[c]Definition of relationship to the intervention
- Unrelated (The AE is clearly NOT related to intervention)
- Unlikely (The AE is doubtfully related to the intervention)
- Possible (The AE may be related to the intervention)
- Probable (The AE is likely related to the intervention)
- Definite (The AE is clearly related to the intervention)
