# Supplementary material for: The safety of co-administration of Bacille Calmette-Guérin (BCG) and influenza vaccines
Source: PLoS One. 2022 Jun 3;17(6):e0268042. doi: 10.1371/journal.pone.0268042 (PMC9165819; doi:10.1371/journal.pone.0268042)
Supplement: S2 Table — Data are presented as n (%) or median (interquartile range), unless otherwise specified. (PDF) [file pone.0268042.s003.pdf]

S2 Table. Local adverse reactions at influenza vaccination site, by prior BCG vaccination history.

Data are presented as n (%) or median (interquartile range), unless otherwise specified.

	**Total Influenza+BCG group** n=1293	**Influenza + BCG-naive group** n=620	**Influenza + BCG-revacc group** n=673	**p-value**
**Pain**	397 (30.7%)	226 (36.5%)	171 (25.4%)	<0.001
None	896 (69.3%)	394 (63.6%)	502 (74.6%)	
Grade 1	342 (26.5%)	197 (31.8%)	145 (21.6%)	0.8
Grade 2	53 (4.1%)	28 (4.5%)	25 (3.7%)	
Grade 3	2 (0.2%)	1 (0.2%)	1 (0.2%)	
Grade 4	0 (0.0%)	0 (0.0%)	0 (0.0%)	
Onset, days	1 (1-1)	1 (1-1)	1 (1-1)	0.04
Mean [SD]	1.2 [0.7]	1.2 [0.6]	1.3 [0.8]	
Duration, days	2 (1-2)	2 (1-2)	2 (1-2)	0.7
**Tenderness**	789 (61.0%)	402 (64.8%)	387 (57.5%)	0.01
None	504 (39.0%)	218 (35.2%)	286 (42.5%)	
Grade 1	537 (41.5%)	259 (41.8%)	278 (41.3%)	0.1
Grade 2	246 (19.0%)	139 (22.4%)	107 (15.9%)	
Grade 3	6 (0.5%)	4 (0.7%)	2 (0.3%)	
Grade 4	0 (0.0%)	0 (0.0%)	0 (0.0%)	
Onset, days	1 (1-1)	1 (1-1)	1 (1-1)	0.6
Duration, days	2 (1-3)	2 (1-3)	2 (1-3)	0.4
**Erythema**	94 (7.3%)	48 (7.7%)	46 (6.8%)	0.5
None	1199 (92.7%)	572 (92.3%)	627 (93.2%)	
Grade 1	89 (6.9%)	46 (7.4%)	43 (6.4%)	0.6
Grade 2	5 (0.4%)	2 (0.3%)	3 (0.5%)	
Grade 3	0 (0.0%)	0 (0.0%)	0 (0.0%)	
Grade 4	0 (0.0%)	0 (0.0%)	0 (0.0%)	
Onset, days	1 (1-2)	1(1-2)	1(1-2)	0.6
Duration, days	2 (1-3)	1 (1-3)	2 (1-3)	0.3
Maximal diameter, cm	1.0 (0.5, 2.8)	1.0 (0.5, 1.5)	1.4 (0.5, 3.0)	0.1
**Swelling**	81 (6.2%)	44 (7.1%)	37 (5.8%)	0.2
None	1212 (93.7%)	576 (92.9%)	636 (94.5%)	
Grade 1	60 (4.6%)	31 (5.0%)	29 (4.3%)	0.4
Grade 2	21 (1.6%)	13 (2.1%)	8 (1.2%)	
Grade 3	0 (0.0%)	0 (0.0%)	0 (0.0%)	
Grade 4	0 (0.0%)	0 (0.0%)	0 (0.0%)	
Onset, days	1 (1-2)	1 (1-2)	1 (1-2)	0.4
Duration, days	2 (1-3)	2 (1.5-3)	2 (1-2)	0.4
Maximal diameter [cm]	2.0 (1.0, 3.0)	1.5 (1.0, 2.5)	2.0 (1.0, 3.0)	0.1
